# Multifocal Cutaneous and Musculoskeletal *Mycobacterium haemophilum* Infection Mimicking Erythema Nodosum and Crystal Arthropathy in a Kidney Transplant Recipient: A Case Report

**DOI:** 10.1155/crit/9038193

**Published:** 2026-07-30

**Authors:** Zoe Behrman, Muhamed Abubacker

**Affiliations:** ^1^ West Virginia School of Osteopathic Medicine, Lewisburg, West Virginia, USA, wvsom.edu; ^2^ Department of Rheumatology, WVU Medicine Thomas Hospitals, Charleston, West Virginia, USA

**Keywords:** crystal arthropathy, erythema nodosum, kidney transplantation, *Mycobacterium haemophilum*, nontuberculous mycobacteria, opportunistic infection

## Abstract

**Background:**

*Mycobacterium haemophilum* is an uncommon nontuberculous mycobacterium that primarily affects immunocompromised hosts and may present with cutaneous and musculoskeletal manifestations that mimic inflammatory or infectious disorders. Its specialized growth requirements often delay diagnosis, particularly in solid organ transplant recipients.

**Case Presentation:**

A 59‐year‐old man with a history of deceased donor kidney transplantation presented with progressive bilateral ankle pain, lower extremity swelling, and painful nodular skin lesions. Initial evaluation suggested erythema nodosum and inflammatory arthritis, with nondiagnostic musculoskeletal imaging and arthrocentesis. Corticosteroid therapy resulted in only transient improvement. Persistent low‐titer serum cryptococcal antigenemia with negative cerebrospinal fluid studies further complicated the diagnostic evaluation and prompted empiric fluconazole therapy. Progressive disease led to recurrent hospitalization and inability to bear weight. Initial synovial fluid aspiration demonstrated acid‐fast bacilli, whereas repeat bilateral aspirations demonstrated concomitant monosodium urate crystals and acid‐fast bacilli. Skin biopsy demonstrated rod‐shaped acid‐fast organisms with negative fungal staining. Routine cultures were unrevealing, but independent polymerase chain reaction testing performed at two reference laboratories identified *M. haemophilum*. Mycophenolate mofetil was withheld because of active infection, whereas tacrolimus therapy was continued with therapeutic drug monitoring. Prolonged multidrug antimicrobial therapy was individualized according to susceptibility testing and medication tolerance. Despite treatment‐related complications, the patient experienced substantial clinical improvement with healing of cutaneous lesions, restoration of mobility, and preservation of kidney allograft function.

**Conclusion:**

This case highlights the diagnostic complexity of *M. haemophilum* infection in kidney transplant recipients. Simultaneous erythema nodosum–like lesions, crystal arthropathy, and persistent serum cryptococcal antigenemia obscured the diagnosis, whereas conventional microbiologic studies remained largely unrevealing. Early consideration of opportunistic nontuberculous mycobacterial infection and the use of molecular diagnostics may facilitate timely diagnosis and appropriate management in immunocompromised patients.

## 1. Introduction

Nontuberculous mycobacteria (NTM) are increasingly recognized opportunistic pathogens in solid organ transplant recipients because of chronic immunosuppression, improved long‐term graft survival, and advances in diagnostic techniques. Although pulmonary disease remains the most common manifestation, extrapulmonary involvement of the skin, soft tissues, joints, and musculoskeletal system is increasingly reported and is associated with substantial morbidity and prolonged treatment [1, 2]. The incidence of NTM infection among solid organ transplant recipients is significantly higher than that of the general population, with kidney transplant recipients representing an important at‐risk population because of chronic immunosuppressive therapy and multiple competing inflammatory and metabolic conditions that may obscure the diagnosis [3, 4].

*Mycobacterium haemophilum* is an uncommon, slow‐growing NTM first recognized as a human pathogen in 1978. Unlike many other mycobacterial species, it requires lower incubation temperatures (30°C–32°C) and iron‐ or hemin‐supplemented media for optimal growth, making recovery by conventional microbiologic methods difficult [5–7]. Consequently, routine bacterial, fungal, and mycobacterial cultures may remain unrevealing despite progressive disease, and diagnosis is frequently delayed. Histopathology, acid‐fast staining, and molecular diagnostic techniques, including polymerase chain reaction (PCR), have become increasingly important for establishing the diagnosis, particularly in immunocompromised hosts [5, 8].

The clinical manifestations of *M. haemophilum* are diverse and include cutaneous nodules, cellulitis‐like lesions, septic arthritis, tenosynovitis, osteomyelitis, and multifocal soft tissue infection [6, 7]. These presentations frequently mimic more common disorders such as erythema nodosum, crystal arthropathy, inflammatory arthritis, vasculitis, and fungal infection. Partial responses to corticosteroid therapy and initially unrevealing microbiologic investigations may further complicate the diagnostic evaluation and delay appropriate treatment. In transplant recipients, management is particularly challenging because prolonged multidrug antimicrobial therapy must be balanced with maintenance immunosuppression to preserve allograft function while minimizing medication‐related toxicities and the risk of rejection [1, 9–11].

Although cutaneous and musculoskeletal *M. haemophilum* infections have been described in solid organ transplant recipients, presentations simultaneously mimicking erythema nodosum and crystal arthropathy remain uncommon [6, 11]. The coexistence of persistent low‐titer serum cryptococcal antigenemia may further complicate the diagnostic evaluation of immunocompromised patients with progressive musculoskeletal and cutaneous disease [12]. We report a kidney transplant recipient with multifocal cutaneous and musculoskeletal *M. haemophilum* infection that initially masqueraded as erythema nodosum and crystal arthropathy and was ultimately diagnosed through histopathologic findings and independent molecular confirmation from two reference laboratories. This case highlights the importance of maintaining a broad differential diagnosis and integrating molecular diagnostic techniques into the evaluation of progressive cutaneous and musculoskeletal disease in transplant recipients [13].

## 2. Case Presentation

A 59‐year‐old man with a history of deceased donor kidney transplantation in 2015 for end‐stage renal disease presented with progressive bilateral ankle pain, lower extremity swelling, and painful nodular skin lesions. His medical history was significant for hypertension and hypothyroidism. Maintenance immunosuppression consisted of tacrolimus, mycophenolate mofetil, and prednisone.

**Figure 1:**
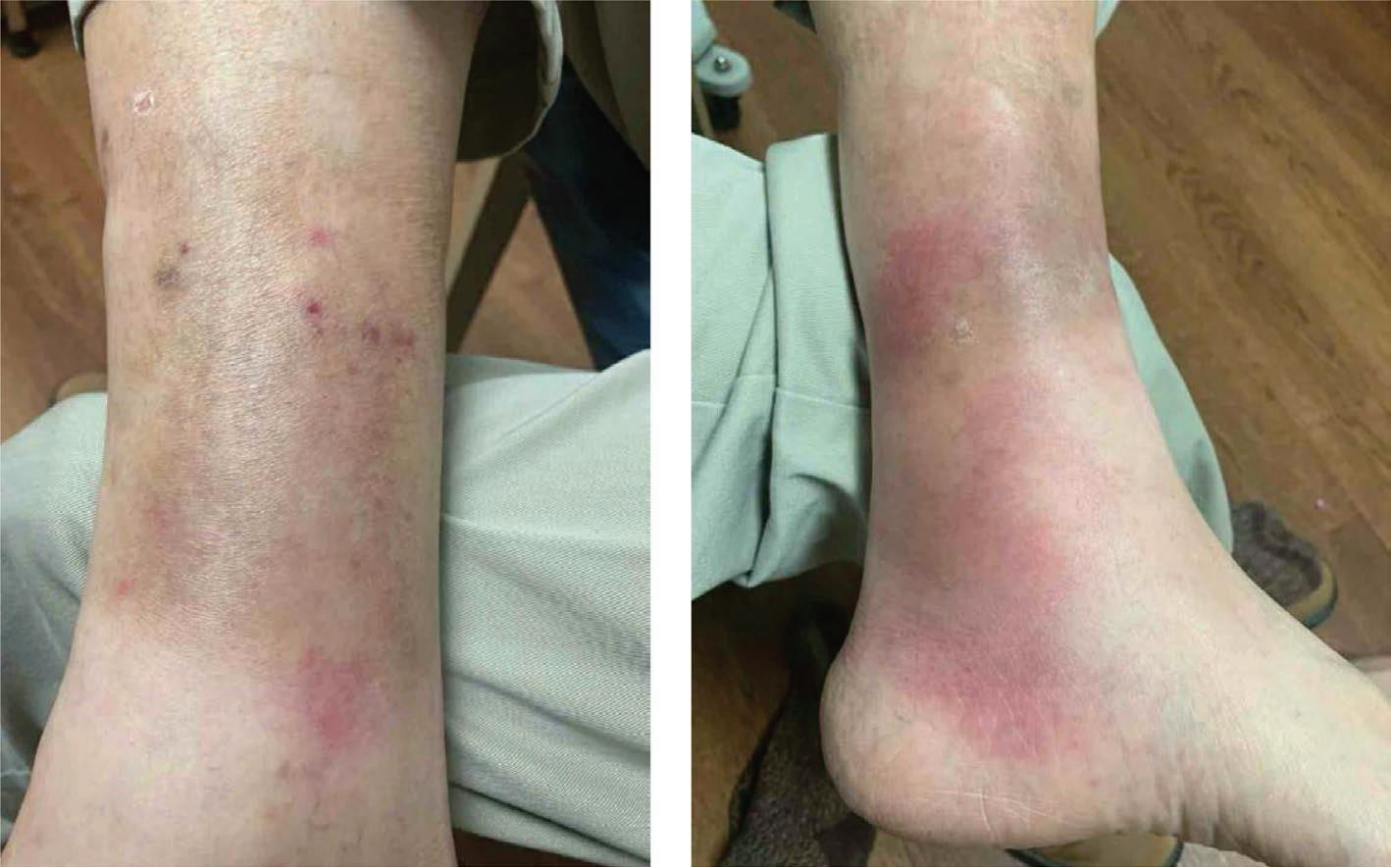
Initial clinical presentation (April 22, 2025). Painful erythematous nodules and plaques involving the bilateral lower extremities at initial rheumatologic evaluation. The lesions were initially interpreted as erythema nodosum–like inflammatory changes and were associated with progressive ankle pain and swelling.

His symptoms began in April 2025 with painful swelling involving both ankles and feet associated with erythematous nodular lesions of the lower extremities. Representative clinical photographs obtained during the initial rheumatology evaluation are shown in Figure 1. He was evaluated by the WVU Medicine Rheumatology service on April 22, 2025. Musculoskeletal ultrasound demonstrated soft tissue swelling without characteristic sonographic features of gout. Skin biopsy demonstrated septal panniculitis with granulomatous inflammation consistent with erythema nodosum, whereas special stains for fungal and acid‐fast organisms were negative. Arthrocentesis did not identify monosodium urate crystals. Because the overall clinical picture favored erythema nodosum with inflammatory arthritis, prednisone was increased with partial improvement of the skin lesions. Colchicine was subsequently initiated because of concern for crystal arthropathy; however, the patient′s symptoms progressively worsened. A comprehensive summary of the chronological diagnostic evaluation, including rheumatologic assessment, musculoskeletal imaging, histopathologic findings, microbiologic investigations, and molecular diagnostic testing, is provided in Table S1.

On May 26, 2025, he presented to Charleston Area Medical Center because of persistent pain and progressive swelling. Serum cryptococcal antigen testing was positive at a low titer by lateral flow assay, whereas cerebrospinal fluid cryptococcal antigen and fungal cultures were negative. There was no evidence of central nervous system or pulmonary cryptococcal disease. Although low‐titer serum cryptococcal antigen results may represent false‐positive findings, particularly at titers of ≤ 1:5, the patient′s immunocompromised state prompted empiric fluconazole therapy while further evaluation continued [14]. No dedicated fungal cultures of synovial fluid or tissue were obtained at this time, which represents a limitation of the initial diagnostic evaluation.

Despite treatment, bilateral foot and ankle swelling progressed, eventually resulting in inability to bear weight and recurrent hospitalization in July 2025. Right ankle synovial fluid aspiration demonstrated acid‐fast bacilli without monosodium urate crystals. Because of progressive cutaneous and musculoskeletal disease, the patient was referred to UPMC Presbyterian Hospital and admitted on July 29, 2025, for further evaluation by the transplant infectious diseases and transplant nephrology services.

**Figure 2:**
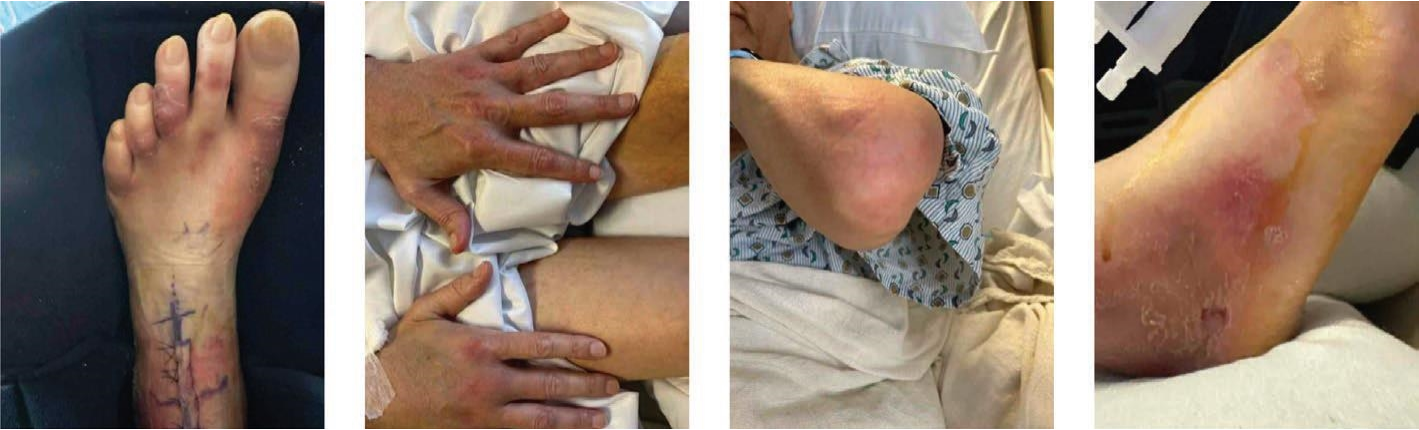
Progressive multifocal cutaneous and musculoskeletal disease (August 3, 2025). Clinical photographs obtained during hospitalization demonstrate extensive multifocal involvement of the upper and lower extremities, including the hands, elbow, ankle, and leg. Marked soft tissue inflammation, nodular cutaneous lesions, and progressive disease burden are evident. Postsurgical changes from diagnostic and therapeutic procedures are visible on the lower extremity.

Physical examination demonstrated bilateral ankle and foot swelling with violaceous nodular skin lesions involving the lower extremities and progressive involvement of the hands and wrists. Representative photographs obtained during hospitalization demonstrate the progression of cutaneous and musculoskeletal disease involving the hands, elbow, ankle, and lower extremity in Figure 2. Blood cultures remained negative, and additional infectious evaluation, including QuantiFERON‐TB testing and Lyme serology, was unrevealing. Serum cryptococcal antigen remained detectable at a low titer.

Magnetic resonance imaging demonstrated bilateral soft tissue edema, ankle joint effusions, and inflammatory changes involving the feet and ankles without abscess formation. Repeat bilateral ankle aspirations demonstrated inflammatory synovial fluid containing concomitant monosodium urate crystals and acid‐fast bacilli. Routine bacterial and fungal cultures remained unrevealing.

Because of progressive cutaneous disease, a left dorsal foot skin biopsy was performed on August 3, 2025. Histopathologic examination demonstrated a dense neutrophilic inflammatory infiltrate with fibrinoid necrosis. Acid‐fast bacilli and Fite stains highlighted rod‐shaped organisms, whereas periodic acid–Schiff staining was negative for fungal elements. Tissue cultures did not identify a pathogen.

Empiric therapy with imipenem, tigecycline, linezolid, and azithromycin was initiated on August 4, 2025, based on the histopathologic identification of acid‐fast organisms, whereas additional microbiologic studies were pending. Synovial fluid aspirated on July 23, 2025, subsequently resulted on August 8, 2025, with PCR testing at the Mayo Clinic reference laboratory identifying *M. haemophilum.* Independent molecular testing performed at the University of Washington reference laboratory on synovial fluid obtained at UPMC also confirmed *M. haemophilum*. No additional efforts were made to recover the organism under specialized culture conditions (hemin‐supplemented media at 30°C–32°C) after molecular identification was established at two independent reference laboratories, which represents a limitation of the microbiologic evaluation [5, 7]. Based on these findings, the patient was started on targeted therapy with rifabutin, levofloxacin, and azithromycin on August 8, 2025. The combination of histopathologic findings, acid‐fast staining, and concordant molecular identification established the diagnosis of multifocal cutaneous and musculoskeletal *M. haemophilum* infection [5, 9].

Management required close collaboration between transplant infectious diseases and transplant nephrology services. Mycophenolate mofetil was withheld because of active infection, whereas tacrolimus therapy was continued with therapeutic drug monitoring [1, 9]. Prednisone dosing was adjusted according to inflammatory manifestations and transplant requirements. Rifabutin was selected over rifampin because of its less potent induction of CYP3A4 and consequently fewer drug–drug interactions with calcineurin inhibitors, as recommended in reviews of NTM management in transplant recipients, although rifabutin can still significantly reduce tacrolimus levels and necessitates close monitoring [10, 11]. Antimicrobial therapy was individualized according to susceptibility testing and medication tolerance and ultimately consisted of a prolonged multidrug regimen including azithromycin, rifabutin, levofloxacin, minocycline, and clofazimine [8, 9]. Fluconazole therapy was continued for approximately 6 months with serial monitoring of serum cryptococcal antigen; it was discontinued in November 2025. After discontinuation, the serum cryptococcal antigen remained low positive and was closely monitored through serial testing. The complete antimicrobial regimen, immunosuppressive modifications, and major therapeutic adjustments throughout the patient′s clinical course are summarized in Table S2.

**Figure 3:**
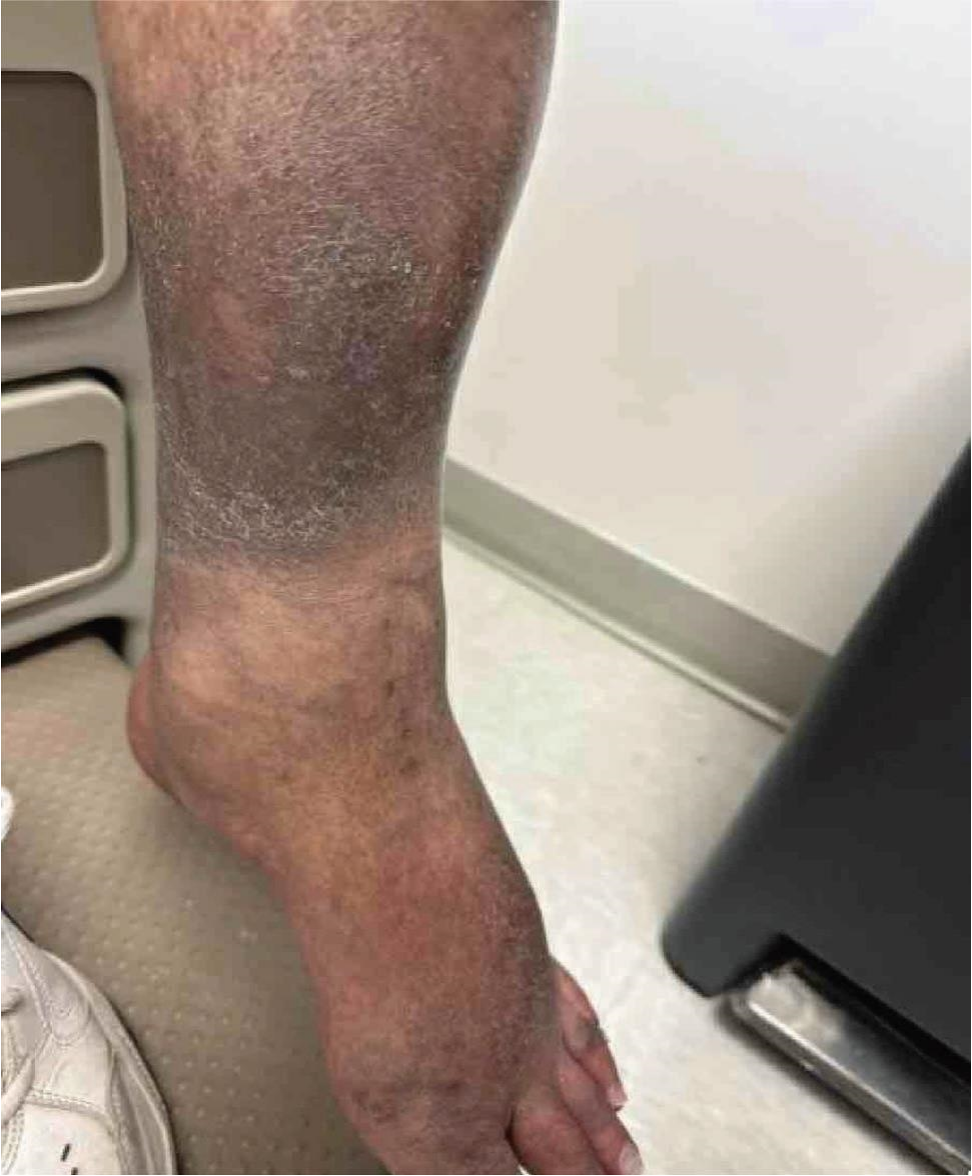
Clinical improvement following prolonged multidrug antimicrobial therapy (January 2026). Follow‐up photographs demonstrate complete healing of previously active cutaneous lesions and ulcerations. Residual chronic lower extremity edema, postinflammatory hyperpigmentation, chronic venous stasis changes, and secondary lymphedema persisted despite resolution of active infection and inflammatory disease.

The clinical course was complicated by acute kidney injury, thrombocytopenia, gastrointestinal intolerance, and weight loss requiring temporary modification of therapy and close outpatient follow‐up. Despite these complications, gradual clinical improvement occurred during prolonged treatment. Draining wounds resolved, cutaneous lesions healed, and pain and swelling progressively improved. The patient regained independent ambulation and returned to normal daily activities. Follow‐up clinical photographs obtained in January 2026 demonstrated substantial improvement compared with the initial presentation in Figure 3. At the most recent follow‐up in May 2026, he remained under the care of the UPMC Transplant Infectious Diseases service, continued prolonged multidrug therapy, and had no open cutaneous wounds despite residual chronic lower extremity edema. The chronology of the patient′s diagnostic evaluation, treatment, and clinical course is summarized in Table 1. A detailed summary of treatment‐related complications, clinical outcomes, and functional recovery during prolonged multidrug antimicrobial therapy is provided in Table S3.

**Table 1:** CARE timeline of clinical events.

Time	Diagnostic/therapeutic activity
2015	Deceased donor kidney transplantation for end‐stage renal disease; maintenance immunosuppression with tacrolimus, mycophenolate mofetil, and prednisone
April 2025	Onset of bilateral ankle pain, lower extremity swelling, and painful nodular skin lesions
April 22, 2025	Evaluation at WVU Medicine Rheumatology; musculoskeletal ultrasound showed soft tissue swelling without sonographic features of gout; skin biopsy demonstrated septal panniculitis with granulomatous inflammation consistent with erythema nodosum; special stains negative for fungal and acid‐fast organisms; arthrocentesis without monosodium urate crystals; prednisone increased
May 2025	Colchicine initiated for suspected crystal arthropathy; symptoms progressively worsened
May 26, 2025	Presentation to Charleston Area Medical Center; serum cryptococcal antigen positive at low titer; CSF cryptococcal antigen and fungal cultures negative; no CNS or pulmonary cryptococcal disease identified; empiric fluconazole initiated
July 2025	Progressive bilateral foot and ankle swelling with inability to bear weight; recurrent hospitalization; right ankle synovial fluid aspiration demonstrated acid‐fast bacilli without monosodium urate crystals
July 23, 2025	Synovial fluid sent for PCR testing at Mayo Clinic reference laboratory
July 29, 2025	Admission to UPMC Presbyterian Hospital; evaluation by transplant infectious diseases and transplant nephrology; bilateral ankle and foot swelling with violaceous nodular skin lesions involving lower extremities, hands, and wrists; MRI demonstrated bilateral soft tissue edema, ankle joint effusions, and inflammatory changes without abscess; repeat bilateral ankle aspirations demonstrated concomitant monosodium urate crystals and acid‐fast bacilli; routine bacterial and fungal cultures unrevealing
August 3, 2025	Left dorsal foot skin biopsy: Dense neutrophilic inflammatory infiltrate with fibrinoid necrosis; acid‐fast bacilli and Fite stains highlighted rod‐shaped organisms; PAS stain negative for fungal elements; tissue cultures negative
August 4, 2025	Empiric therapy initiated with imipenem, tigecycline, linezolid, and azithromycin based on histopathologic AFB identification
August 8, 2025	PCR from Mayo Clinic reference laboratory identified *Mycobacterium haemophilum*; independent molecular testing at University of Washington reference laboratory also confirmed *M. haemophilum*; targeted therapy initiated with rifabutin, levofloxacin, and azithromycin; mycophenolate mofetil withheld
August–December 2025	Prolonged multidrug antimicrobial therapy with azithromycin, rifabutin, levofloxacin, minocycline, and clofazimine; tacrolimus continued with therapeutic drug monitoring; prednisone adjusted; fluconazole continued with serial cryptococcal antigen monitoring; clinical course complicated by acute kidney injury, thrombocytopenia, gastrointestinal intolerance, and weight loss requiring temporary modification of therapy
November 2025	Fluconazole discontinued after approximately 6 months of therapy with serial negative cryptococcal antigen monitoring
January 2026	Follow‐up clinical photographs demonstrated substantial improvement; draining wounds resolved; cutaneous lesions healed; pain and swelling improved
May 2026	Most recent follow‐up; continued under care of UPMC Transplant Infectious Diseases; prolonged multidrug therapy ongoing; no open cutaneous wounds; residual chronic lower extremity edema; independent ambulation restored; kidney allograft function preserved

## 3. Discussion

*M. haemophilum* is an uncommon opportunistic nontuberculous mycobacterium that primarily affects immunocompromised individuals, including solid organ transplant recipients, patients receiving corticosteroids, and those with advanced immunodeficiency. Because the organism requires lower incubation temperatures and iron‐ or hemin‐supplemented media for growth, conventional microbiologic investigations may fail to establish the diagnosis, resulting in delayed recognition and treatment [5–7].

Several aspects of the present case illustrate the diagnostic challenges associated with *M. haemophilum* infection. The patient′s initial presentation with painful nodular skin lesions and inflammatory arthritis closely resembled erythema nodosum. Histopathology demonstrated septal panniculitis with granulomatous inflammation, and early special stains were negative for fungal and acid‐fast organisms. Partial improvement following corticosteroid therapy further supported an inflammatory diagnosis. Similar presentations have been described in previous reports of *M. haemophilum* infection in transplant recipients, where cutaneous manifestations including plaques, nodules, and chronic ulcers predominantly affect cooler acral areas of the body and may initially be attributed to inflammatory or autoimmune conditions [6, 11]. These findings emphasize that opportunistic infections should remain in the differential diagnosis of transplant recipients with persistent cutaneous and musculoskeletal disease despite apparently reassuring initial investigations.

An additional diagnostic challenge was the coexistence of crystal arthropathy. Initial arthrocentesis did not demonstrate monosodium urate crystals; however, repeat bilateral aspirations demonstrated concomitant monosodium urate crystals and acid‐fast bacilli. Hyperuricemia and crystal‐induced arthritis are common among kidney transplant recipients because of chronic kidney disease and calcineurin inhibitor therapy [12]. The coexistence of crystal arthropathy and septic arthritis is well recognized; Papanicolas et al. reported that 5% of joints with crystal arthritis had concomitant infection and that crystals alone in synovial fluid from acute monoarthritis cannot exclude septic arthritis [13]. This case further demonstrates that crystal arthropathy and opportunistic infection may coexist and should not be considered mutually exclusive, particularly in immunocompromised patients.

Persistent low‐titer serum cryptococcal antigenemia further complicated the diagnostic evaluation. Although cerebrospinal fluid studies and the evaluation for invasive cryptococcal disease were negative, the patient′s immunocompromised state prompted empiric antifungal therapy while additional investigations were pursued. Dizon et al. demonstrated that low serum cryptococcal antigen titers (≤ 1:10) correlated with cryptococcal disease in a substantial proportion of non‐HIV immunocompromised patients, supporting the clinical decision to initiate antifungal therapy, while also noting that false‐positive results occur at low titers and require careful clinical correlation [14]. In our patient, tacrolimus trough levels were closely monitored throughout fluconazole therapy and consistently remained within the therapeutic range. In retrospect, persistent cryptococcal antigenemia represented an important diagnostic confounder rather than the primary explanation for the patient′s progressive musculoskeletal and cutaneous manifestations. This experience highlights the importance of reassessing the differential diagnosis when clinical progression continues despite apparently plausible explanations.

The microbiologic findings in this case emphasize the importance of integrating histopathology with modern molecular diagnostics. Routine bacterial and fungal cultures remained unrevealing, whereas skin biopsy demonstrated acid‐fast organisms and independent PCR testing performed at two reference laboratories identified *M. haemophilum*. Previous reports have similarly demonstrated that molecular techniques, including PCR targeting the 16S rRNA gene and the *M. haemophilum*–specific internal transcribed spacer, may establish the diagnosis even when conventional cultures are nondiagnostic [5, 7]. Early consideration of specialized microbiologic testing may reduce diagnostic delay and facilitate earlier initiation of appropriate therapy.

Management of *M. haemophilum* infection in transplant recipients remains challenging because there are no standardized treatment guidelines. The American Society of Transplantation Infectious Diseases Community of Practice recommends macrolide‐based combination therapy for slow‐growing NTM, including *M. haemophilum*, with additional agents including fluoroquinolones and rifamycins [9]. Shah et al. reviewed NTM infections after solid organ transplantation and emphasized the importance of prolonged multidrug therapy, careful selection of antimicrobial agents to minimize drug–drug interactions with immunosuppressive medications, and individualized reduction of immunosuppression based on infection severity and rejection risk [10]. Nookeu et al. reported that combination therapy with macrolides and fluoroquinolones resulted in a 60% cure rate for cutaneous *M. haemophilum* infection, with the addition of a rifamycin as a third drug for more severe cases yielding a modest 66% cure rate [8]. Yasen et al., in a literature review of 79 cases with cutaneous involvement, found that triple therapy with a quinolone, macrolide, and rifamycin was most commonly used in clinical practice, with an overall good prognosis, although iatrogenic immunosuppression and dissemination were associated with worse outcomes [6]. In our patient, treatment required multiple regimen adjustments because of medication‐related toxicities and therapeutic monitoring.

Equally important was the careful management of immunosuppression. Mycophenolate mofetil was withheld because of active infection, whereas tacrolimus therapy was continued with close monitoring and prednisone dosing was adjusted according to inflammatory manifestations and transplant requirements. Reduction of immunosuppression is a recognized component of NTM management in transplant recipients, and Shah et al. recommended that the degree of reduction should be individualized based on the severity of infection, risk of allograft rejection, and time since transplantation [1, 9, 10]. Rifabutin was selected over rifampin because of its less potent induction of CYP3A4, as highlighted by Shah et al. in their review of drug–drug interactions between antimycobacterial agents and immunosuppressive medications in transplant recipients [10]. Although rifabutin has fewer interactions than rifampin, it can still significantly reduce tacrolimus levels, necessitating close therapeutic drug monitoring [10, 11]. Successful management required close collaboration among transplant infectious diseases, transplant nephrology, rheumatology, pathology, and local providers.

An important strength of this case is the availability of longitudinal follow‐up demonstrating treatment response. The patient′s longitudinal clinical course, including serial clinical assessments, resolution of cutaneous disease, preservation of kidney allograft function, and ongoing multidisciplinary follow‐up, is summarized in Table S4. The patient experienced gradual healing of cutaneous lesions, improvement in pain and swelling, restoration of independent ambulation, and preservation of kidney allograft function despite a prolonged and complicated clinical course. The availability of both initial and follow‐up clinical photographs further demonstrates the substantial response achieved with individualized multidisciplinary care. In the literature review by Jurairattanaporn et al., infection resolved completely in 80% of transplant patients with *M. haemophilum*, with treatment duration varying from 3 weeks to 15 months [7].

This case has several important clinical implications. First, *M. haemophilum* infection should be considered in transplant recipients with persistent cutaneous lesions and inflammatory arthritis, particularly when initial investigations are unrevealing or responses to corticosteroid therapy are incomplete [6–8]. Second, concomitant conditions such as crystal arthropathy and persistent cryptococcal antigenemia may coexist and complicate diagnostic reasoning rather than fully explain the patient′s presentation [13, 14]. Third, molecular diagnostic techniques should be considered early when fastidious opportunistic pathogens are suspected, and direct communication with microbiology laboratory personnel is encouraged to promote appropriate culture techniques [5, 7]. Finally, successful treatment requires balancing prolonged antimicrobial therapy with maintenance immunosuppression and careful multidisciplinary follow‐up to preserve allograft function while controlling infection [1, 9, 10].

## 4. Conclusion

*M. haemophilum* infection should be considered in the differential diagnosis of kidney transplant recipients with persistent cutaneous lesions and inflammatory arthritis, particularly when the clinical course is progressive and routine microbiologic investigations are unrevealing. This case demonstrates how multifocal cutaneous and musculoskeletal infection may mimic erythema nodosum and crystal arthropathy, whereas persistent serum cryptococcal antigenemia can further complicate the diagnostic evaluation.

Definitive diagnosis required integration of clinical findings, histopathology, acid‐fast staining, and independent molecular confirmation from two reference laboratories. Successful management depended on prolonged individualized multidrug antimicrobial therapy, careful adjustment of immunosuppression, and close multidisciplinary collaboration among transplant infectious diseases, transplant nephrology, rheumatology, pathology, and local providers.

This case highlights the importance of maintaining a broad differential diagnosis in immunocompromised patients with progressive musculoskeletal and cutaneous disease and supports the early use of molecular diagnostic techniques when fastidious opportunistic pathogens are suspected [15]. Early recognition and coordinated multidisciplinary management may improve outcomes while preserving allograft function in this vulnerable population.

### 4.1. Clinical Learning Points

- M. haemophilum should be considered in kidney transplant recipients with persistent cutaneous lesions and inflammatory arthritis that fail to respond to conventional therapy [6–8].
- Opportunistic infection and crystal arthropathy may coexist, and the presence of monosodium urate crystals should not exclude an underlying infectious process [13].
- Persistent low‐titer serum cryptococcal antigenemia may complicate the diagnostic evaluation of immunocompromised patients and should not preclude investigation for alternative opportunistic infections [14].
- Routine microbiologic studies may be unrevealing because of the specialized growth requirements of M. haemophilum; histopathology and molecular diagnostic techniques can facilitate earlier diagnosis [5–7].
- Successful management requires prolonged multidrug antimicrobial therapy, careful adjustment of immunosuppression, and close multidisciplinary collaboration to control infection while preserving allograft function [1, 9, 10].

## Author Contributions

**Zoe Behrman:** writing – original draft. **Muhamed Abubacker:** writing – review and editing, formal analysis, supervision.

## Funding

No funding was received for this manuscript.

## Ethics Statement

Institutional review board approval was not required for this single‐patient case report in accordance with institutional policy. Written informed consent for publication was obtained from the patient.

## Consent

Written informed consent was obtained from the patient for publication of this case report and the accompanying clinical images. The patient underwent deceased donor kidney transplantation in accordance with accepted institutional protocols and applicable federal regulations governing organ procurement and allocation. Donor consent for organ donation was obtained through the organ procurement organization at the time of donation, consistent with standard practice under the Uniform Anatomical Gift Act. No donor‐identifiable information is included in this report, and no additional donor consent for publication was required or obtainable.

## Conflicts of Interest

The authors declare no conflicts of interest.

## Patient Perspective

The patient experienced significant frustration related to the prolonged diagnostic process and the delay in establishing appropriate treatment for his condition. Progressive pain, swelling, and cutaneous lesions substantially impaired mobility and daily activities, resulting in multiple hospitalizations and prolonged medical therapy. Although the patient experienced gradual clinical improvement with multidisciplinary care and individualized antimicrobial treatment, the illness and its treatment resulted in lasting sequelae, including chronic lower extremity venous stasis changes. Despite these persistent symptoms, the patient achieved healing of the cutaneous lesions, improved functional mobility, and preservation of kidney allograft function and continues close follow‐up with the transplant infectious diseases team.

## Supporting information


**Supporting Information** Additional supporting information can be found online in the Supporting Information section. Table S1: The chronological diagnostic evaluation. Table S2: Antimicrobial and immunosuppressive management. Table S3: Treatment‐related complications and clinical outcomes. Table S4: Longitudinal follow‐up.

## Data Availability

The data supporting the findings of this case report are available from the corresponding author upon reasonable request. Additional clinical information has been omitted to protect patient confidentiality.
