# Supplementary material for: Multifocal Cutaneous and Musculoskeletal Mycobacterium haemophilum Infection Mimicking Erythema Nodosum and Crystal Arthropathy in a Kidney Transplant Recipient: A Case Report
Source: Case Rep Transplant. 2026 Jul 30;2026:9038193. doi: 10.1155/crit/9038193 (PMC13424453; doi:10.1155/crit/9038193)
Supplement: Supplementary file 1 — Supporting Information Additional supporting information can be found online in the Supporting Information section. Table S1: The chronological diagnostic evaluation. Table S2: Antimicrobial and immunosuppressive management. Table S3: Treatment‐related complications and clinical outcomes. Table S4: Longitudinal follow‐up. [file CRIT-2026-9038193-s001.docx]

**Supplementary Material**

## Supplementary Table 1. Chronology of Diagnostic Evaluation

| **Date** | **Investigation** | **Key Findings** |
| --- | --- | --- |
| April 22, 2025 | WVU Rheumatology evaluation | Bilateral ankle and foot swelling with painful nodular skin lesions. |
| April 2025 | Musculoskeletal ultrasound | Soft tissue swelling without characteristic sonographic features of gout. |
| April 2025 | Skin biopsy | Septal panniculitis with granulomatous inflammation consistent with erythema nodosum; fungal and AFB stains negative. |
| April 2025 | Arthrocentesis | No monosodium urate crystals identified. |
| May 26, 2025 | Serum cryptococcal antigen | Positive. |
| May 2025 | Cerebrospinal fluid studies | Cryptococcal antigen and fungal cultures negative. |
| July 2025 | Right ankle aspiration | Acid-fast bacilli identified. |
| July/August 2025 | MRI lower extremities | Bilateral soft tissue inflammation and ankle joint involvement without abscess formation. |
| August 2025 | Bilateral ankle aspirations | Monosodium urate crystals and acid-fast bacilli identified. |
| August 3, 2025 | Left dorsal foot biopsy | Acid-fast organisms identified; PAS negative for fungal organisms. |
| August 8, 2025 | Mayo Clinic PCR | Mycobacterium haemophilum identified. |
| August 2025 | University of Washington molecular testing | Independent confirmation of M. haemophilum. |

## Supplementary Table 2. Immunosuppression and Antimicrobial Management

| **Therapy** | **Clinical Course** |
| --- | --- |
| Tacrolimus | Continued with therapeutic drug monitoring throughout treatment. |
| Mycophenolate mofetil | Withheld because of active infection. |
| Prednisone | Dose adjusted according to inflammatory manifestations and transplant requirements. |
| Fluconazole | Initiated after positive serum cryptococcal antigen and continued for approximately six months. |
| Azithromycin | Included in prolonged multidrug therapy for M. haemophilum. |
| Rifabutin | Selected as part of multidrug therapy with transplant specialist oversight. |
| Levofloxacin | Added according to susceptibility profile and clinical response. |
| Minocycline | Incorporated into prolonged antimicrobial regimen. |
| Clofazimine | Added during long-term treatment. |
| Therapy modifications | Medication adjustments required because of toxicity and clinical tolerance. |

## Supplementary Table 3. Clinical Course and Treatment-Related Complications

| **Complication** | **Clinical Outcome** |
| --- | --- |
| Progressive inflammatory arthritis | Improved with targeted antimicrobial therapy. |
| Cutaneous lesions | Gradual healing with resolution of draining wounds. |
| Acute kidney injury | Improved following supportive care and treatment adjustment. |
| Thrombocytopenia | Managed with modification of therapy. |
| Gastrointestinal intolerance | Required medication adjustments. |
| Weight loss | Improved during recovery. |
| Functional impairment | Progressed to inability to bear weight before diagnosis; subsequently regained independent ambulation. |
| Chronic lower extremity venous stasis changes | Persisted despite resolution of active infection. |
| Kidney allograft function | Preserved during prolonged treatment. |

## Supplementary Table 4. Longitudinal Follow-up

| **Follow-up** | **Clinical Status** |
| --- | --- |
| January 2026 | Marked improvement in skin lesions, inflammatory arthritis, and mobility documented during WVU Medicine Rheumatology follow-up. |
| January 2026 | Clinical photographs demonstrated substantial healing compared with initial presentation. |
| May 2026 | Continued follow-up with UPMC Transplant Infectious Diseases and WVU Medicine Rheumatology. |
| May 2026 | No open cutaneous wounds. |
| May 2026 | Stable kidney allograft function. |
| May 2026 | Continued prolonged multidrug antimicrobial therapy. |
| May 2026 | Residual chronic lower extremity venous stasis changes present. |
